# The nature and extent of emotion recognition and empathy impairments in children showing disruptive behaviour referred into a crime prevention programme

**DOI:** 10.1007/s00787-019-01358-w

**Published:** 2019-06-01

**Authors:** Laura M. Hunnikin, Amy E. Wells, Daniel P. Ash, Stephanie H. M. van Goozen

**Affiliations:** 1 School of Psychology, Cardiff University, Cardiff, Wales UK; 2 Department of Clinical Child and Adolescent Studies, Leiden University, Leiden, The Netherlands; 3 Department of Criminology and Criminal Justice, University of Northampton, Northampton, UK

**Keywords:** Disruptive behaviour, Emotion recognition, Empathy, Eye gaze

## Abstract

Childhood disruptive behaviour has been linked to later antisocial and criminal behaviour. Emotion recognition and empathy impairments, thought to be caused by inattention to the eye region, are hypothesised to contribute to antisocial and criminal behaviour. This is the first study to simultaneously examine emotion recognition and empathy impairments, their relationship, and the mechanism behind these impairments, in children with disruptive behaviour. We hypothesised that children with disruptive behaviour would exhibit negative emotion recognition and cognitive and affective empathy impairments, but that these impairments would not be due to reduced attention to the eye region. We expected these emotion impairments to be driven by disruptive behaviour. We also expected a relationship between emotion recognition and cognitive empathy only. Ninety-two children with disruptive behaviour, who were participating in a police crime prevention programme and rated by their schoolteacher using the Strengths and Difficulties Questionnaire (DB; mean age 8.8 years, 80% male), took part. There was a comparison group of 58 typically developing children (TD; mean age 9.7 years, 78% male). All children completed emotion recognition and empathy tasks, both with concurrent eye tracking to assess social attention. Not only were DB children significantly impaired in negative emotion and neutral emotion recognition, and in cognitive and affective empathy compared to the TD children, but severity of disruptive behaviour also predicted intensity of emotion impairments. There were no differences in social attention to the eye region. Negative emotion recognition and empathy impairments are already present in an identifiable group of children displaying disruptive behaviour. These findings provide evidence to encourage the use of targeted interventions.

## Key points

**Table:** 

The study shows that children with disruptive behaviour are impaired in negative emotion and neutral face recognition and in cognitive and affective empathy but not in social attention.
Emotion recognition and cognitive and affective empathy impairments were not related.
Emotion recognition and empathy impairments were driven by severity of disruptive behaviour over and above the influence of IQ and age.
Early and targeted interventions that tackle emotion-specific impairments in children who display disruptive behaviour should be considered.

## Introduction

Antisocial behaviour (ASB) describes a persistent pattern of negative behaviours [1], which has costly consequences for the individual and society [2–4]. Two mechanisms are important in explaining the behavioural characteristics of ASB: emotion recognition and empathy impairments.

Antisocial individuals are typically impaired in recognising expressions of fear and sadness [5]. However, impairments in other emotions have been identified, including anger [6], general negative emotions [7], and all basic emotions [8].

Empathy is the ability to understand and share another’s emotional state. Cognitive empathy is the cognitive awareness of another’s emotional state and affective empathy is the vicarious experience of another’s emotions [9]. Some studies have shown that antisocial individuals are impaired in cognitive and affective empathy [10]; others found evidence for impaired affective but intact cognitive empathy [11]. The findings for cognitive empathy are less consistent than for affective empathy. Different definitions and operationalizations of cognitive empathy are likely to contribute to these discrepant findings [12].

Emotion recognition impairments are thought to underlie impaired empathy [13, 14]. Models of empathy generally assume a three-stage approach [13, 15], involving recognising the emotion in another, taking their perspective and then feeling an emotional response. Empathy has been related to emotion recognition accuracy across basic emotions [16], recognition accuracy at lower intensities [17], and fear recognition [18]. It has been shown that facial emotion recognition was positively correlated with cognitive but not affective empathy [19].

Attention to the eyes is a mechanism thought to be responsible for both emotion recognition and empathy abilities. Dadds and colleagues showed that boys with ASB plus callous-unemotional (CU) traits showed normal patterns of fear recognition when directing their attention to the eye region [20]. Research has also shown a positive relationship between empathy and looking towards the eyes during emotional clips [21]. However, recent evidence with at-risk children, adolescents with Conduct Disorder (CD), or adolescents with Attention Deficit Hyperactivity Disorder (ADHD) plus CD have shown that attention to the eye region does not account for emotion recognition and empathy impairments [11, 22, 23].

Both emotion recognition and empathy are important mechanisms in the development and continuation of ASB. Distress cues, such as facial expressions of fear and sadness, are thought to possess perceptual cues that elicit empathy [24]. The ability to empathise with others inhibits ASB, because empathic people find their own negative behaviour vicariously punishing [13]. Distressing facial expressions also serve as social reinforcers that condition developing children to avoid engaging in behaviours that elicit these expressions in others. An inability to understand distressing cues means that an individual cannot use them to adapt their behaviour in a socially appropriate manner and stimulus-reinforcement learning is inhibited. This is specified by the Integrated Emotion Systems model [14], which was initially developed for psychopathy, but has since been applied to ASB more broadly.

One of the strongest predictors of later criminal and antisocial behaviour is disruptive behaviour in childhood [25, 26]. However, despite emotion recognition and empathy impairments being involved in criminal and antisocial behaviour, no study has yet simultaneously investigated these impairments, their relationship, and the mechanism behind these impairments in children with disruptive behaviour.

This study investigated emotion recognition and empathy impairments in children showing disruptive behaviour, as described by their schoolteacher. Their performance on emotion recognition and empathy tasks was compared to a sample of typically developing children. Concurrent eye tracking was conducted during both emotion recognition and empathy tasks to gain a better understanding of the mechanism behind these impairments, namely social attention to the eyes.

This study had the following hypotheses. First, we predicted that children with disruptive behaviour would show negative emotion recognition impairments and cognitive and affective empathy impairments. However, we predicted that affective empathy impairments would be limited to negative emotions only in comparison to the typically developing children. Second, we expected that emotion recognition impairments would be related to cognitive, but not affective, empathy impairments. Third, based on recent research findings, we predicted that emotion recognition and empathy impairments would not be related to impaired social attention to the eyes. Finally, we predicted that the more severe the disruptive behaviour, the more intense the emotion recognition and empathy impairments.

## Method

### Participants

The 164 participants (119 male) aged 7–11 years who took part in this study were categorised into two groups according to the behaviours which they displayed. One participant group showed disruptive behaviour (DB group). The other group formed a typically developing comparison group (TD group).

The 106 children assigned to the DB group were showing disruptive behaviour and participating in a police crime prevention programme, called the Early Intervention Hub, developed by Northamptonshire Police Force. The Hub was set up to address the high number of children displaying problematic behaviour in the county [27] and aims to provide support to at-risk families whose children show disruptive behaviour, ultimately aiming to play a preventative role via early intervention. As part of the Hub, a Police Community Support Officer (PCSO) is placed in the child’s school to support at-risk children. Referrals into the Hub are done through Police Protection Notices, referring professionals (schools, police officers, and social workers) and through Early Help co-ordinators. Typically, the children referred to the Hub have been subjected to a wide range of Adverse Childhood Experiences, including poverty, mental health issues within the home, and domestic abuse. Because of these factors, the children have been classified by the police as being at high risk for future negative outcomes, including criminal and antisocial behaviour. The children have no formal mental health diagnosis, and because they have not yet reached a crisis point, they are considered “the blind spot” of the social services [28].

Children in the DB group were referred to participate in this study from the Hub PCSO working at the child’s school. After referral, the children’s teachers completed the Strengths and Difficulties Questionnaire (SDQ) [29] to confirm disruptive behaviour status. To be included in the DB group, an SDQ score in the ‘slightly raised’ or above range for conduct or peer problems (≥ 3) or ‘slightly lowered’ or below range for prosocial behaviour (≤ 5) was required. This terminology and cut-off scores are from the SDQ scoring and represent just 10% of the UK population [30]. Participants only needed to reach the threshold for one of the three SDQ subscales to be eligible.

The hyperactivity subscale of the SDQ was not chosen as a recruitment criterion, because recent studies have shown that emotion recognition impairments in ADHD are specific to those with comorbid CD [23]. Similarly, the emotion problem subscale was not used as this is related to internalising problems and emotion recognition impairments are more related to externalising problems [31, 32]. Inclusion criteria for the DB group were, therefore, a referral from the Hub and showing an elevated or lowered score on the previously described SDQ subscales.

A comparison group of 58 typically developing children participated. Parent-completed SDQ scores confirmed TD group status (within ‘close to average’ range for total difficulties; a score of ≤ 13/40). Inclusion criteria were a referral from school teachers for not showing disruptive behaviour and meeting the SDQ criteria previously described.

Exclusion criterion for both groups was an estimated IQ (intelligence quotient) less than 70 and not completing the tasks within the study. Based on these criteria, 14 children were excluded from the DB group, leaving a final sample of 92 DB children and 58 TD children.

### Materials

#### Demographic and behavioural characteristics

The Wechsler Abbreviated Scale of Intelligence [33] provided an estimated IQ score. Socioeconomic status (SES) was estimated using Office for National Statistics estimates of average household weekly income based on postcode (low = £0–£520; middle = £521–£670; high = £671+).

The SDQ is a 25-item widely used, valid, and reliable questionnaire assessing problematic and prosocial behaviour [29].

#### Facial emotion recognition

The Facial Emotion Recognition (FER) [7] test consists of 60 photos of male and female faces of varying ethnicities and ages displaying four emotions (happiness, sadness, fear, and anger) plus a neutral expression from the Radboud Faces Database [34]. Children were asked to choose which emotion the person was displaying. See supplementary information for data collected during the development and validation of this task.

#### Empathy

Participants viewed three clips from Harry Potter films, which evoked empathic reactions. Each clip represented happiness, sadness, or fear as agreed upon by 96% of 31 6-to-11-year old children in a preliminary study. The previous experience with each clip revealed no effect of film familiarity. Participants were asked questions about the main character’s emotions in the clip (cognitive empathy) and their own emotions while viewing the clip (affective empathy). They were asked how strongly they and the main character felt eight emotions and to explain the reason for the emotion. Responses were coded by two individuals using the Cardiff Empathy Scoring System [11, 35, 36]. Cognitive empathy scores ranged from 0 to 9 and affective empathy scores from 0 to 7, with higher scores indicating greater empathy. Interrater reliability between two raters ranged from 0.94 (cognitive) to 0.98 (affective). Fourteen participants (9 TD, 5 DB) were unable to complete the empathy task.

#### Eye tracking

During both emotion recognition and empathy tasks, social attention was examined using concurrent eye tracking. A portable Tobii X2-60 compact eye-tracker sampling at 60 Hz with a screen resolution of 1920 × 1080 was used. Participants were positioned 60 cm away from a 15″ laptop screen. Calibration quality was checked and repeated if necessary. An I-VT fixation filter with a minimum fixation criterion of 60 ms sampled average raw data of both eyes to produce information on eye position and duration. Eye-gaze validity was checked for all recordings using a percentage score of successfully recorded data. Validity ranged from 60 to 99% (mean emotion recognition accuracy: 82%; mean empathy accuracy: 87%). Not all children were able to complete eye tracking during the emotion recognition (*n *= 96; TD: *n *= 43; DB: *n* = 53) or empathy (*n* = 75; TD: *n *= 32; DB: *n *= 43) tasks.

### Procedure

All parts of the study were completed at the child’s school. Children completed the research session, lasting approximately 75 min, with a trained researcher. All participants first provided assent, then completed the FER, empathy, and WASI tasks. During both FER and empathy tasks, concurrent eye tracking was recorded. Participants were then debriefed.

### Statistical analyses

Demographic characteristics were analysed using independent-samples *t* tests for continuous variables, Mann–Whitney *U* test for SES, and Pearson Chi-square test for gender.

Independent-samples *t* tests were used to understand group differences for emotion recognition and cognitive and affective empathy. Analyses were run separately for each emotion and separately for cognitive and affective empathy. Where there were violations in assumptions, a Mann–Whitney *U* test was used instead.

Spearman’s correlations were used to understand the relationship between emotion recognition and cognitive and empathy variables. The full sample was included (both DB and TD) and Bonferroni corrections were applied.

Tobii Studio analysed eye gaze; areas of interest (AOIs) were created around the eyes, mouth, total face, and entire screen. For emotion recognition, eye gaze was analysed during a three-second segment when the face was presented without emotion options. For empathy, eye gaze was analysed during the six-to-eight-second segment that presented the most intense emotional content in each clip. Percentage dwell time to the eyes was calculated by summing all fixations to the eyes divided by the total duration of time spent looking at the face. A two-way ANOVA was run with emotion as the within-subjects factor and group as the between-subjects factor.

Effect sizes were calculated as partial eta squared ($\eta_{\text{p}}^{2}$) for ANOVAs and Cohen’s *d* for *t* test [37]. Confidence intervals are reported for significant findings using parametric tests only.

Multiple regression analyses were carried out to assess the relative contribution of demographic (IQ and age) and behavioural variables (total SDQ score) in explaining any between-group differences in emotion recognition and empathic abilities. Separate multiple regressions were run for negative emotion recognition, cognitive empathy, and affective empathy, with all emotions combined to reduce multiple testing. A stepwise regression model was used with total SDQ entered first followed by age and IQ simultaneously.

## Results

### Demographic and behavioural data

Participants in the TD group were older and had a higher IQ and SES than the DB group (Table 1). The gender ratio was similar for both groups and there was no effect of gender on emotion recognition accuracy (*t*(148) = − 0.86, *p* = 0.39, *d *= 0.18), cognitive empathy (*t*(134) = − 1.50 *p* = 0.14, *d *= 0.31), or affective empathy *(t*(134) = − 1.35, *p* = 0.18, *d *= 0.29). TD participants showed fewer conduct and peer problems and more prosocial behaviour than the DB children did.

**Table 1:** Demographic and behavioural characteristics of participants

Variable	TD (*n *= 58)	DB (*n *= 92)	Value	*p* value	95% confidence interval
Age (years)	9.67 (1.11)	8.82 (1.20)	*t *= − 4.39	< 0.001	− 1.24, − 0.471
IQ	104.65 (17.20)	91.62 (12.72)	*t *= − 4.74	< 0.001	− 18.50, − 7.56
Gender			*χ*^2^ = 0.17	0.68	–
% Male	77.6	80.4			
% Female	20.7	19.6			
SES			*U* = 2932	< 0.001	–
% Low	0	7.3			
% Medium	22	56.1			
% High	78	36.6			
SDQ total	7.49	17.94	*t *= 13.11	< 0.001	8.87, 12.03

Means are presented with standard deviations in brackets. Statistical tests: independent-samples *t* test, Mann–Whitney *U* test for SES and Pearson Chi-square for gender

*IQ* intelligence quotient, *SES* socioeconomic status, *SDQ* Strengths and Difficulties Questionnaire

### Emotion recognition

**Fig. 1:**
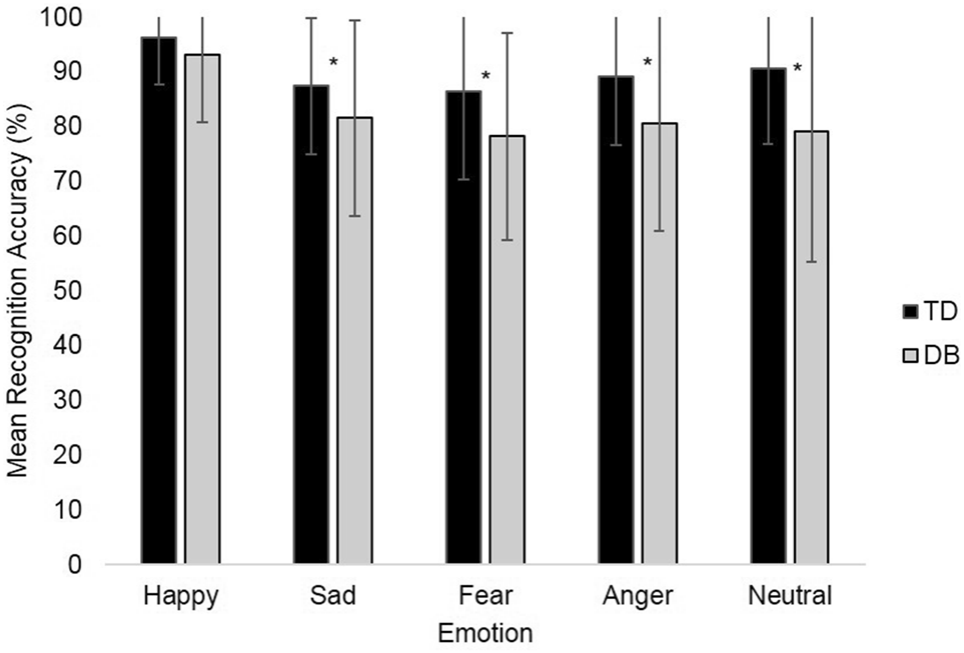
Mean emotion recognition scores. Error bars are set at ± 1 standard deviation. *p ≤ 0.05. Statistical test: independent-samples *t* test

DB participants had significantly lower scores for recognising expressions of sadness (*t*(148) = − 2.18, *p* = 0.03, *d* = 0.38, 95% CI [− 11.13, − 0.54]), fear (*t*(148) = -2.72, *p *= 0.007, *d* = 0.47, 95% CI [− 14.10, − 2.26]), anger (*t*(147.99) = -3.25, *p* = 0.001, *d* = 0.52, 95% CI [− 13.75, − 3.36]), and neutral (*t*(146.99) = − 3.67, *p* < 0.001, *d* = 0.58, 95% CI [− 17.46, − 5.24]) (see Fig. 1). There was no group difference for recognition of happiness expressions (*t*(146.66) = − 1.84, *p* = 0.07, *d* = 0.30).

### Empathy

**Fig. 2:**
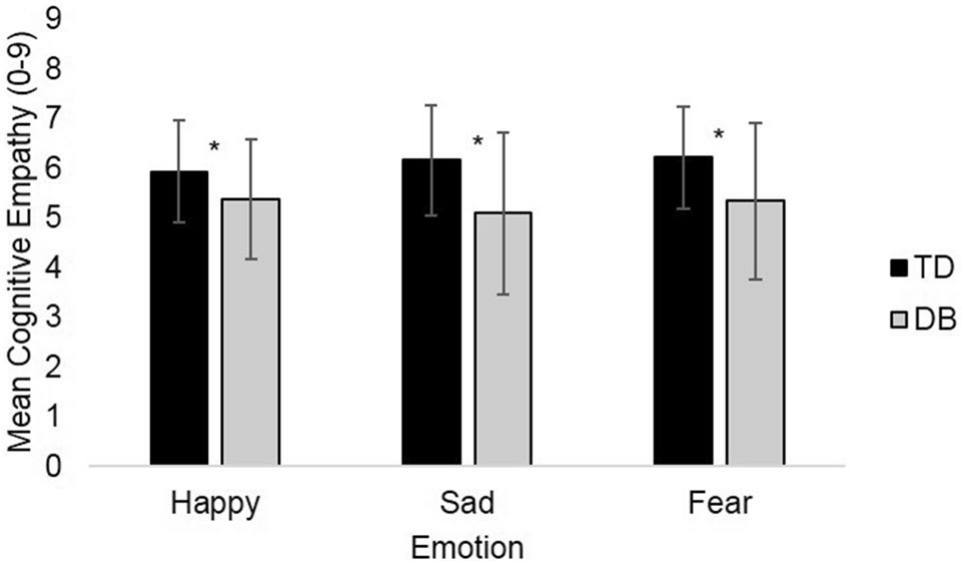
Mean cognitive empathy scores. Error bars are set at ± 1 standard deviation. *p ≤ 0.05. Statistical test: independent-samples *t* test

**Fig. 3:**
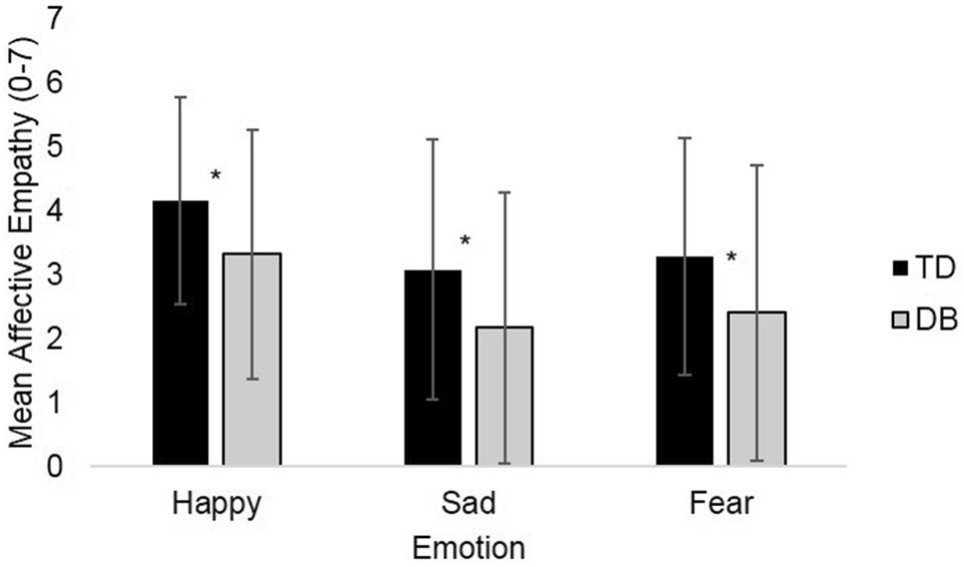
Mean affective empathy scores. Error bars are set at ± 1 standard deviation. *p ≤ 0.05. Statistical test: independent-samples *t* test

DB participants scored significantly lower on cognitive empathy than TD participants for happiness (*t*(134) = − 2.80, *p* = 0.006, *d *= 0.51, 95% CI [− 0.97, − 17]), sadness (*t*(129.59) = − 4.53, *p* < 0.001, *d *= 0.76, 95% CI [− 1.54, − 0.60]), and fear (*t*(131.11) = − 3.93, *p* < 0.001, *d *= 0.66, 95% CI [− 1.32, − 0.44]) (Fig. 2). The same group differences were observed for affective empathy; the DB group scored significantly lower than the TD group for happiness (*t*(134) = − 2.56, *p* = 0.012, *d *= 0.47, 95% CI [− 1.49, − 0.19]), sadness (*t*(134) = − 2.42, *p* = 0.02, *d *= 0.44, 95% CI [− 1.65, − 0.17]), and fear (*t*(117.97) = − 2.40, *p* = 0.018, *d *= 0.42, 95% CI [− 1.59, − 0.15]) (Fig. 3).

### Relationships between emotion recognition accuracy and empathy

There were no significant relationships between emotion recognition accuracy and cognitive or affective empathy (Table 2).

**Table 2:** Relationships between emotion recognition and empathy variables

	Happy ER	Sad ER	Fear ER	Anger ER	Neutral ER
Happiness CE	0.04	0.02	0.02	0.10	0.09
Sadness CE	0.19	0.10	0.15	0.18	0.11
Fear CE	0.09	0.08	0.08	0.13	0.01
Happiness AE	0.00	0.07	− 0.10	0.19	− 0.09
Sadness AE	0.12	0.18	0.05	0.09	0.07
Fear AE	0.04	0.08	− 0.09	0.06	− 0.01

Full sample (both DB and TD): *N* = 136. Statistical test: Spearman’s correlation. Bonferroni corrections applied

*ER* emotion recognition, *CE* cognitive empathy, *AE* affective empathy

### Eye tracking: Dwell time to the eye region

**Fig. 4:**
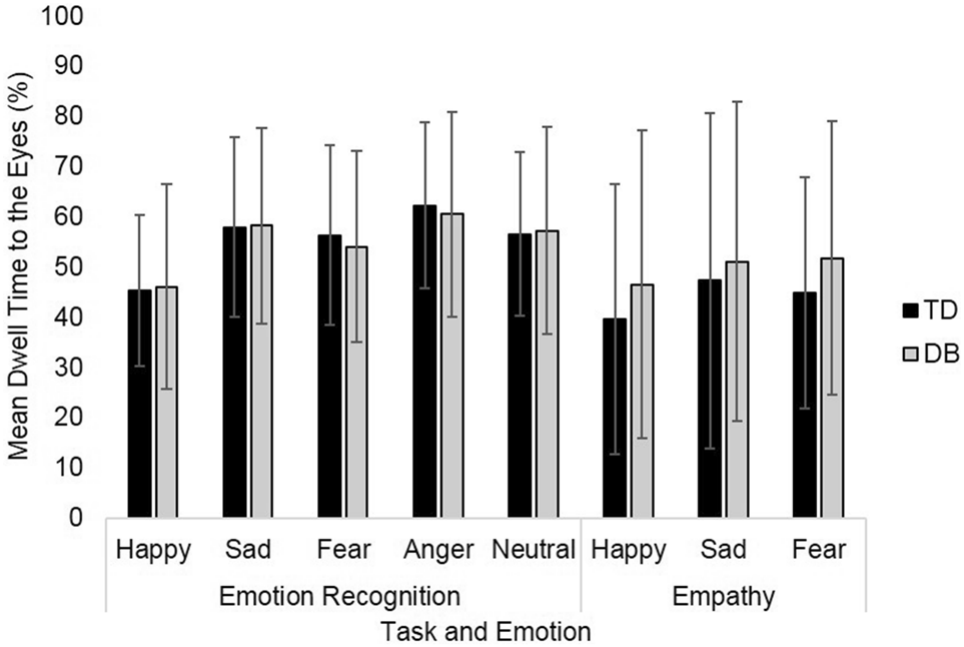
Mean percentage dwell time to the eyes for emotion recognition and empathy tasks. Error bars at set at ± 1 standard deviation. Statistical test: one-way ANOVA

The two groups did not differ in attention to the screen during the emotion recognition [*t*(94) = − 1.97, *p* = 0.05, *d *= 0.40] and empathy tasks [*t*(73) = − 0.92, *p* = 0.36, *d *= 0.22]. There was no group difference in dwell time to the eyes during either the emotion recognition *(F*(1, 94) = 0.02, *p* = 0.90, $\eta_{\text{p}}^{2}$ = 0.00) or empathy task (*F*(1, 62) = 1.06, *p* = 0.31, $\eta_{\text{p}}^{2}$ = 0.02) (Fig. 4).

### Predictors of impaired emotion recognition and empathy

As there were no group differences in happiness recognition or social attention, regressions were not run for these variables. Given its importance in disruptive behaviour, overall negative expression recognition was included in the multiple regression for emotion recognition.

*Emotion recognition* The full model of age, IQ, and total SDQ was statistically significant, *R*^2^ = 0.132, *F*(3, 127) = 6.46, *p* < 0.001, adj. *R*^2^ = 0.112. The addition of age and IQ to the prediction of emotion recognition did not lead to a statistically significant change in *R*^2^ of 0.03, *F*(2, 127) = 2.17, *p* = 0.12. Full details of all regression models are presented in Table 3.

**Table 3:** Multiple regression predicting emotion recognition, cognitive empathy, and affective empathy from SDQ, age, and IQ

Variable	Emotion recognition				Cognitive empathy				Affective empathy			
	Model 1		Model 2		Model 1		Model 2		Model 1		Model 2	
	*B*	*β*	*B*	*β*	*B*	*β*	*B*	*β*	*B*	*β*	*B*	*β*
Constant	91.21		64.06		6.04		3.90		3.47		3.03	
SDQ	− 0.63**	− 0.32	− 0.47*	− 0.24	− 0.04*	− 0.29	− 0.03*	− 0.19	− 0.04*	− 0.18	− 0.04	− 0.18
Age			0.94	0.08			0.11	0.15			0.05	0.04
IQ			0.17	0.18			0.01	0.16			− 0.001	− 0.01
*R*^2^	0.10		0.13		0.08		0.10		0.03		0.04	
*F*	14.77**		6.46**		10.55*		5.19*		4.05*		1.40	

Model 1 = SDQ (strengths and difficulties) total score. Model 2 = SDQ, IQ and age. Emotion recognition *N* = 131. Empathy *N *= 119 *p < 0.05, **p < 0.001. *B* = unstandardized regression coefficient. *β *= standardised coefficient

*Cognitive empathy* The full model of age, IQ, and total SDQ was statistically significant, *R*^2^ = 0.119, *F*(3, 115) = 5.19, *p* = 0.002, adj. *R*^2^ = 0.096. The addition of age and IQ to the model did not lead to a statistically significant change in *R*^2^ of 0.04, *F*(2, 115) = 2.38, *p* = 0.10 (see Table 3).

*Affective empathy* The full model of age, IQ, and total SDQ was not statistically significant, *R*^2^ = 0.04, *F*(3, 115) = 1.40, *p* = 0.248, adj. *R*^2^ = 0.010. However, model 1 with just total SDQ score was significant, *R*^2^ = 0.033, *F*(1, 117) = 4.05, *p* = 0.047, adj.*R*^2^ = 0.025 (see Table 3).

## Discussion

This study is the first to show that similar emotion recognition and empathy impairments as have been found in antisocial and criminal adults are evident in a sample of younger children who are concurrently displaying disruptive behaviour and are taking part in a crime prevention programme. Interestingly, their emotion and empathy impairments were unrelated and not caused by impaired attention to the eyes.

We hypothesised that children with disruptive behaviour would show more emotion recognition impairments than typically developing children. Our hypothesis was supported as the DB group demonstrated problems in recognition of negative (sad, fear, and anger) and neutral expressions [8]. These findings provide evidence to encourage the use of early interventions to address emotional problems before they become entrenched.

We hypothesised that children with disruptive behaviour would show also cognitive and affective empathy impairments compared to the typically developing group, and that affective empathy impairments would be specific to negative emotions. Our hypotheses were largely confirmed and were in line with the previous studies [10, 38]. However, for affective empathy, we did not hypothesise that these children would also show impaired affective empathy for happiness based on the previous studies [11, 39]. Future research should aim to clarify the nature of affective empathy impairments in antisocial populations, especially as happiness may diffuse hostility and encourage prosocial behaviour [40].

This study also aimed to understand the relationship between emotion recognition and empathy impairments, hypothesising that there would be a relationship between emotion recognition and cognitive, but not affective empathy, based on the study by Lui and colleagues [19]. Contrary to expectations, we found no evidence for a consistent relationship between emotion recognition and either cognitive or affective empathy. Variations in methodology may be responsible for divergence in findings to the literature showing a relationship between emotion recognition and cognitive empathy; Lui et al. used questionnaires to measure trait empathy [19]; we used affective clips to assess state empathy. To show cognitive empathy, an ability to understand vocal, gestural, and contextual information was required, whereas emotion recognition required an ability to recognise static facial expressions. It is possible that one recognises facial expressions, but struggles with these additional elements required for cognitive empathy [41]. Research shows that antisocial individuals are impaired in vocal [42] and postural emotion recognition [43] and struggle to integrate multiple sources of emotional information [44].

As hypothesised, problems in social attention did not explain performance on the emotion recognition and empathy tasks. This finding contrasts with Dadds et al. [20], but confirms recent evidence in at-risk children and adolescents with ADHD and CD [11, 23]. Indeed, evidence in typically developing individuals has also shown no relationship between emotion recognition and social attention to the eyes [45]. Given that our groups differed in emotion recognition but not in social attention, the interpretation of facial features in antisocial individuals requires more research.

We predicted that emotion recognition and empathy impairments would be influenced primarily by disruptive behaviour. Regression analyses confirmed that severity of disruptive behaviour uniquely predicted emotion recognition, and cognitive and affective empathy impairments, over and above the influence of IQ and age. These findings support the idea that emotion recognition and empathy abilities are important in disruptive behaviour, thus, further providing evidence for the importance of early and targeted interventions.

Our study had some limitations. First, practical limitations prevented the collection of eye tracking data in the full sample and eye movement behaviour was considered across all trials, regardless of accuracy. It is possible that eye movement behaviours would be different when considering correct versus incorrect trials. Some children may have had difficulties verbalising their thoughts and feelings [46] and future research should, therefore, employ physiological measures to examine empathy in young samples. In addition, because the DB group only had to reach the threshold for one of three of the SDQ subscales and because the children were exposed to a range of different risk factors (e.g., domestic abuse and mentally ill parents), it is possible that these factors could have influenced their emotion recognition and empathy abilities. For example, Pollak and Sinha [47] showed that physically abused children require less sensory input to identify facial displays of anger than controls. Future research should aim to examine the influence of these risk factors on emotion recognition and empathy ability. In addition, we did not consider the role of hyperactivity scores on emotion recognition abilities. Whilst some research has identified that emotion recognition is not related to ADHD [23]. However, other research has provided alternative findings [48]. Future research should investigate the influence of hyperactivity on emotion recognition, empathy, and social attention. It is important to note that our groups did not differ in attention to the screen during the eye tracking. Finally, this study did not consider the influence of CU traits despite their hypothesised importance in emotion recognition and empathy [10, 20]. Future research should investigate the role of CU traits in the emotion recognition and empathy abilities of children with disruptive behaviour.

### Conclusions and clinical implications

Disruptive behaviour in childhood is related to later antisocial and criminal behaviour. We have provided evidence that emotion recognition impairments in antisocial and criminal populations are already present in young children who display disruptive behaviour and, indeed, that severity of disruptive behaviour predicted intensity of emotion impairments. The findings from this research support the use of early interventions that improve emotion recognition and empathy development in these children [49]. A targeted early intervention approach is likely to be more effective and represents a better use of finances and resources [32, 50].
